# Management of Steroid-Resistant Nephrotic Syndrome in Children

**DOI:** 10.7759/cureus.19363

**Published:** 2021-11-08

**Authors:** Sanjana Sachdeva, Syeda Khan, Cristian Davalos, Chaithanya Avanthika, Sharan Jhaveri, Athira Babu, Daniel Patterson, Abdullah J Yamani

**Affiliations:** 1 Medicine, Kasturba Medical College, Mangalore, IND; 2 Medicine and Surgery, Dow University of Health Sciences, Karachi, PAK; 3 Pediatrics, Universidad de las Americas, Quito, ECU; 4 Medicine and Surgery, Karnataka Institute of Medical Sciences, Hubli, IND; 5 Pediatrics, Karnataka Institute of Medical Sciences, Hubli, IND; 6 Internal Medicine, Smt. NHL Municipal Medical College (MMC), Ahmedabad, IND; 7 Pediatrics, Saudi German Hospital, Dubai, ARE; 8 Medicine, Tarumanagara University, Jakarta, IDN; 9 Pediatric Medicine, Coast General Teaching and Referral Hospital, Mombasa, KEN

**Keywords:** pediatrics, steroid resistance, nephrosis, hypoalbuminemia, nephrotic syndrome, steroids

## Abstract

Nephrotic syndrome (NS) affects 115-169 children per 100,000, with rates varying by ethnicity and location. Immune dysregulation, systemic circulating substances, or hereditary structural abnormalities of the podocyte are considered to have a role in the etiology of idiopathic NS. Following daily therapy with corticosteroids, more than 85% of children and adolescents (often aged 1 to 12 years) with idiopathic nephrotic syndrome have full proteinuria remission. Patients with steroid-resistant nephrotic syndrome (SRNS) do not demonstrate remission after four weeks of daily prednisolone therapy. The incidence of steroid-resistant nephrotic syndrome in children varies between 35 and 92 percent. A third of SRNS patients have mutations in one of the important podocyte genes. An unidentified circulating factor is most likely to blame for the remaining instances of SRNS.

The aim of this article is to explore and review the genetic factors and management of steroid-resistant nephrotic syndrome. An all language literature search was conducted on MEDLINE, COCHRANE, EMBASE, and Google Scholar till September 2021. The following search strings and Medical Subject Headings (MeSH) terms were used: “Steroid resistance”, “nephrotic syndrome”, “nephrosis” and “hypoalbuminemia”. We comprehensively reviewed the literature on the epidemiology, genetics, current treatment protocols, and management of steroid-resistant nephrotic syndrome.

We found that for individuals with non-genetic SRNS, calcineurin inhibitors (cyclosporine and tacrolimus) constitute the current mainstay of treatment, with around 70% of patients achieving full or partial remission and an acceptable long-term prognosis. Patients with SRNS who do not react to calcineurin inhibitors or other immunosuppressive medications may have deterioration in kidney function and may develop end-stage renal failure. Nonspecific renal protective medicines, such as angiotensin-converting enzyme inhibitors, angiotensin 2 receptor blockers, and anti-lipid medications, slow the course of the illness. Recurrent focal segmental glomerulosclerosis in the allograft affects around a third of individuals who get a kidney transplant, and it frequently responds to a combination of plasma exchange, rituximab, and increased immunosuppression. Despite the fact that these results show a considerable improvement in outcome, further multicenter controlled studies are required to determine the optimum drugs and regimens to be used.

## Introduction and background

Nephrotic syndrome (NS) is the most common glomerular disease in children. It is characterized as proteinuria (nephrotic range) with serum albumin less than 3 g/dl or clinical evidence of edema when albumin level cannot be done [1]. It affects 4.7 per 100,000 children worldwide with varying ethnic differences [2]. One of the studies done over 12 years found that the incidence of NS was higher in South Asian children than those of European descent [3].

The mainstay of treatment has been prednisone for the past 50 years, yet the exact mechanism of the disease is still not precise. Cyclophosphamide, calcineurin inhibitors (CNI), rituximab, and mycophenolate mofetil are often used to induce or maintain remission [2]. NS is classified based on the response to glucocorticoid treatment as steroid-sensitive nephrotic syndrome (SSNS) or steroid-resistant nephrotic syndrome (SRNS). SRNS is defined as a condition of non-remission after four weeks of daily prednisone therapy at a dose of 2 mg/kg per day. SRNS, which accounts for 20% of the total cases, is more likely to be associated with focal segmental glomerulosclerosis (FSGS) [4-5]. It is known that 36-50% of SRNS cases will progress to end-stage renal failure within 10 years [6].

The management of SRNS remains a challenge for pediatric nephrologists, as there is no predictor for resistance to steroid therapy. In the past few years, several genetic mutations essential for podocyte function have been identified, which may play a significant role in understanding molecular mechanisms of glomerular filtration. The first evidence of a genetic cause of congenital NS came with the discovery of the NPHS1 and NPHS2 genes. Since then, over 45 genes have been found, accounting for 20-30% of familial NS [7]. Because the cases of SRNS with genetic mutations are not immune-mediated, they are theoretically unresponsive to immunosuppressants also [8].

Due to the low number of cases of SRNS per year, there has been a lack of evidence-based algorithms for the treatment. Due to the large number of genetic variations involved, the treatment of SRNS entails trying and testing a number of treatment options, including newer, upcoming drugs. To understand and interpret the currently available evidence, we conducted a review on the management of SRNS in children.

## Review

Epidemiology

The incidence of nephrotic syndrome in the pediatric population is one to two per 100,000 children as reported by a New Zealand study [9-10], among which 80% are steroid-sensitive and 20% are steroid-resistant [5]. Steroid-resistant nephrotic syndrome (SRNS) accounts for almost 15% of all children with chronic kidney disease who eventually require renal transplantation [11]. Sadowski CE et al. studied 1783 unrelated families and found that single-gene mutations caused SRNS in 29.5% of cases, out of which 25.3% of children were aged one to six years, 17.8% of children were aged from seven to 12 years, and 10.8% were adolescents aged 13 to 18 years. They have identified that hereditary podocytopathies are responsible for up to 30% of children with SRNS by genetic screening [12].

Also, it is found that as the age of onset of nephrotic syndrome is increased, the chances of establishing a genetic diagnosis will be reduced [13]. Long-term outcomes of SRNS patients were analyzed by Trautmann A et al. and they found:

a) Diagnosis of genetic disease and end-stage renal disease (ESRD) risk (10 and 15 years ESRD-free survival rates): 27% and 17% for patients with a genetic disease while patients with sporadic disease have 53% and 48%, respectively.

b) Familial disease without a genetic diagnosis had better 10-year renal survival than patients with a genetic diagnosis.

c) Histopathology and ESRD risk (at 5 years and 10 years ESRD-free survival rates): 92% and 79% for minimal change disease, 69% and 52% for focal segmental glomerulosclerosis (FSGS), and 80% at five years for diffuse mesangial sclerosis (DMS) [14].

After renal transplant due to ESRD, about 1/3rd of children develop recurrence of the disease. The studies show that this recurrence of diseases post-transplant is less seen in SRNS with a genetic cause. It is seen in patients with an immunological cause of SRNS [15].

Pathophysiology of steroid-resistant nephrotic syndrome

**Figure 1:**
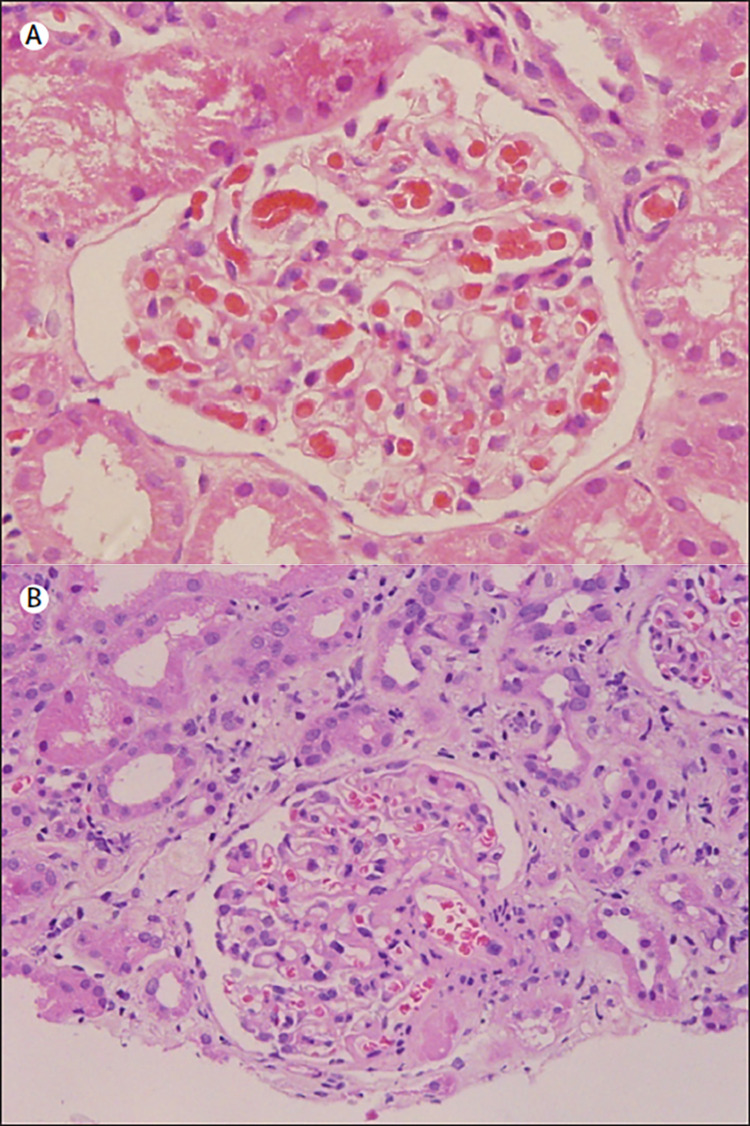
Image of a kidney biopsy taken from a patient with SRNS Normal glomerulus in minimal change disease (A), focal segmental glomerulosclerosis (B). Perihilar segmental sclerosis with hyalinosis, tubular atrophy, and interstitial fibrosis should be noted. Image reproduced with permission [18]. Reprinted from "Management of steroid-resistant nephrotic syndrome in children and adolescents", by Tullus K, Webb H, Bagga A., 2018, Lancet Child Adolesc Health, 2(12):880-890

According to a report from the International Study of Kidney Disease in Children (ISKDC), focal segmental glomerulosclerosis (FSGS) biopsy results were found in 47.5% of children who were non-responders to steroids [16]. Histologically, FSGS is distinguished by sclerosis and podocyte foot process effacement in a few capillary segments of a subset of glomeruli [17]. In addition, there are histopathological variants of minimal change disease (MCD) associated with non-responders, namely, mesangial hypercellularity and focal tubular changes [16]. The glomerular histological finding is normal in minimal change disease while focal segmental glomerulosclerosis will show perihilar segmental sclerosis with hyalinosis, tubular atrophy, and interstitial fibrosis (Figure 1) [18].

Genetic mutations play a big role in podocyte regulation that could cause SRNS, namely, genes that signal the podocyte cleft membrane (NPHS1 Adhesion Molecule, Nephrin (NPHS 1), NPHS2 Stomatin Family Member, Podocin (NPHS2), CD2 Associated Protein (CD2AP), and Protein Tyrosine Phosphatase Receptor Type O (PTPRO)/Glomerular Epithelial Protein-1 (GLEPP1)), regulation of actin assembly (Actinin Alpha 4(ACTN4) and Inverted Formin 2 (INF2)), or glomerular basement membrane interactions (Laminin Subunit Beta 2 (LAMB2) and Integrin Subunit Alpha 2 (ITGA2)) [18-19]. In addition, recessive mutations of the renal ankyrin protein (KANK) also play an essential role in maintaining renal nephron function through the regulation of Rho GTPase activity [20]. Mutations in these genes (NPHS2, alpha-actinin 4 (ACTN4), Phospholipase C Epsilon (PLCE1), and Transient Receptor Potential Cation Channel Subfamily C Member 6 (TRPC6)) leads to the formation of FSGS, which is the most common form in SRNS patients [21-23].

Mutations in the NPHS2 gene cause a recessive form of SRNS, found in 40% of cases of familial SRNS and 6-17% of sporadic SRNS. Patients generally develop SRNS by age six and reach end-stage renal disease (ESRD) in the first decade [21].

The ACTN4 gene, encoding alpha-actinin 4, is a gene that is mainly found in podocytes and slightly in the renal vascular system. Alpha actinin 4 is responsible for the cross-linking of actin filaments. Mutations in this gene lead to a decrease in actin binding, the formation of intracellular aggregates, and a shortened protein half-life. A typical symptom in a patient with an ACTN4 mutation is proteinuria in adolescence and ESRD progression to the fifth decade of life [22].

PLCE1 gene mutation causes arrest of normal glomerular development. A recessive PLCE variant was identified in 28.6% of patients with diffuse mesangial sclerosis and 18% of patients with SRNS [23].

Transient receptor potential cation channel 6 (TRPC6) encodes a calcium channel in the podocyte membrane that regulates mechanosensation and intracellular calcium concentration. TRPC6 variant mutations were found in 12% of children with early-onset and sporadic SRNS [24].

In general, genetic testing is necessary for patients less than six years of age at diagnosis, with a positive family history of nephrotic syndrome, a history of steroid resistance, FSGS histopathology, or diffuse mesangial sclerosis [25].

In addition to genetic defects, the immune system also has a role in SRNS. The nuclear factor kappa-light-chain-enhancer of activated B-cells (NF-kB) is part of the Rail family with two subunits (p50 and p65). Glucocorticoids are known to inhibit inflammation by affecting NF-kB transcription. SRNS patients tend to have lower levels of the NF-kB p65 subunit than partial responders. In-vitro studies have also shown that only cells possessing a complete p65-p50 heterodimer or p60 homodimer can respond to glucocorticoids. If only the p50 subunit is present, resistance to glucocorticoids is formed. Another study showed that SRNS patients experienced a shift of T helper-1 to T helper-2 cytokines, and higher IgE levels were associated with shorter remission times and were more prone to relapse [26].

Genetics

It has been postulated that mutations in genes encoding podocyte-associated proteins are associated with about 30% of cases of SRNS in children [15,27]. They play a role in cell-cell signaling at the podocyte slit membrane (NPHS1 (Nephrin), NPHS2 (Podocin), CD2AP (CD2 associated protein), and PTPRO (protein tyrosine phosphatase receptor type O)/GLEPP1 (glomerular epithelial protein 1)), regulation of foot process actin network (ACTN4 (Alpha- actinin-4 gene) and INF2 (inverted formin 2 gene)), or foot process-glomerular basement membrane interaction (LAMB2 (Laminin B2), and ITGA3 (Integrin alpha 3)) [19]. The genes were studied by Sanger sequencing traditionally, which is now being replaced by whole-exome sequencing or whole-genome sequencing by NGS (next-generation sequencing) methods.

Why Is Genetic Testing Needed in SRNS?

Studies have proved that monogenic SRNS cases show higher resistance to immunosuppressive therapy [8]. So mutation detection allows the physician to avoid these medications and prevent the potential side effects. A personalized treatment approach and avoidance of renal biopsy is an added benefit. Moreover, monogenic SRNS has a better prognosis for post-transplant recurrences. Genetic disease allows screening for other associated medical conditions, especially in syndromic SRNS. It allows for the affected families' genetic counseling, risk stratification, and prenatal diagnosis [28].

Indications of genetic testing include congenital or infantile-onset NS(nephrotic syndrome), childhood-onset NS, family history of NS, consanguinity, and extrarenal manifestations [28].

Genes Implicated in SRNS

SRNS can be inherited as an autosomal recessive, autosomal dominant, or mitochondrial inheritance. More than 50 genes have been identified recently in the pathogenesis of SRNS. Most of them have FSGS as histopathology [28].

Some of the syndromic steroid-resistant nephrotic syndromes are Denys-Drash syndrome and Frasier syndrome, Pierson’s syndrome, nail-patella syndrome, Schimke immuno-osseous dysplasia, mitochondrial encephalomyopathy, lactic acidosis, and stroke-like episodes (MELAS), Charcot-Marie-Tooth disease, and Mandibuloacral dysplasia [28].

Some of the monogenic SRNS are:

1. NPHS1 (nephrin):** **This gene encodes for nephrin and is found to be the cause of congenital nephrotic syndrome and autosomal recessive Finnish type nephrotic syndrome. It causes massive proteinuria and rapid progression to ESRD [29].

2. NPHS2 (Podocin): This gene mutation is attributed to most of the cases of SRNS in infancy (4 to 12 months of age) and during childhood. It encodes for Podocin [30]. It occurs in 40% of familial and 6-17% of sporadic SRNS cases [31]. The age of onset can be from birth to six years and progress to ESRD by the end of the first decade [21]. It was also found that in the Japanese, Chinese, and Korean populations this mutation is found at a low rate [13,32].

p.R229Q variant of NPHS2: This variant has been found recently in juvenile or adult-onset NS. it has been reported in Caucasians, particularly in Europeans (frequency 0.03-0.13) [33]. Extensive research is being conducted about this variant in different populations and frequency has been established according to ethnicity. Although the frequency is varying in different populations, this variant is found to increase the risk of microalbuminuria [34-35].

3. PLCE1 (phospholipase C epsilon-1 gene): In phospholipase C epsilon-1 gene mutation, diffuse mesangial sclerosis histopathology is seen. The patients present early, severe diseases that progress to ESRD [23]. It can manifest at any time from birth to childhood.it is inherited as an autosomal recessive form and affects cell adhesion in the podocyte [23].

4. WT1 (Wilms tumor 1 gene):** **It encodes for Wilms tumor 1, which is very important for the development of the kidney. It is associated with both isolated and syndromic SRNS. Genotype-phenotype correlations from studies show an early onset severe form with DMS and late-onset, slow progression with FSGS [36]. The syndromic associations of the WT1 gene are Fraiser syndrome, Denys-Drash syndrome, and WAGR syndrome [36]. Most of them show urogenital abnormalities and malignancies.

5. TRPC6 (transient receptor potential cation channel 6): This gene encodes for a transient receptor potential cation channel. It plays a role in intracellular calcium signaling. It is inherited as an autosomal dominant form and causes FSGS. The age of onset is late adolescence or early adulthood [12,27,37]. A Turkish study documents the presence of TRPC6 mutations in the population [38] while a south Indian study couldn’t provide statistical evidence for the presence of this mutation in the study population [39]. Recently Italian research suggested that TRPC6 variants were also detected in early-onset and sporadic SRNS cases [40].

6. ACTN4 (alpha-actinin-4 gene): This encodes alpha-actinin-4 and causes late-onset FSGS, and it causes slow progression to ESRD [41-42]. Alpha actinin-4 is responsible for cross-linking of actin filaments in the slit diaphragm. mutations disrupt the cytoskeletal structure of podocytes. Feng D et al. discovered three different point mutations in ACTN4 in three unrelated families with FSGS. They showed autosomal dominant inheritance with incomplete penetrance [43].

7. INF 2 (inverted formin 2 gene): Another gene mutation causing autosomal dominant SRNS. It codes for inverted formin 2. The age of onset is from adolescence to adulthood. It usually causes isolated FSGS, a subgroup of patients is associated with Charcot-Marie-Tooth neuropathy.

8. LAMB2 (laminin subunit β2) and LMX1B (LIM homeobox transcription factor 1β): These mutations are also causing syndromic SRNS. They cause Pierson syndrome and nail-patella syndrome, respectively [44]. It has been also seen in isolated congenital and childhood SRNS.

9. COQ2 (coenzyme Q2) deficiency: It is caused by mutations in genes that encode proteins in the coenzyme Q10 biosynthesis pathway, which is a component in the mitochondrial respiratory chain [45]. It can present as mitochondrial disease or isolated cases. The mutations include COQ2, COQ6 (Coenzyme Q6), PDSS2 (Prenyl-diphosphate synthase subunit 2), ADCK4, etc. [46-47]. These mutations detected patients can be treated with COQ10 supplementation and can modify disease progression [47-48]. ADCK4 occurs during adolescence with proteinuria and advanced CKD. Renal biopsies show FSGS.

10. ACE (angiotensin-converting enzyme) I/D gene polymorphism: Angiotensin-converting enzyme (ACE) causes the conversion of angiotensin I to angiotensin II, which acts in the renin-angiotensin system [49]. This ACE is involved in the pathogenesis of many renal diseases. The ACE gene consists of an insertion allele [I] and a deletion allele [D] forming three possible genotypes - II, ID, and DD [50]. Recently, many studies suggested that there is an association between ACE gene polymorphism and steroid responsiveness in nephrotic syndrome. Some studies [51] showed the association of the D allele to steroid resistance, but some others [52] couldn't find a statistically significant result. More research in all populations is needed in this area to have a proper conclusion.

11. ARHGDIA (Rho GDP dissociation inhibitor alpha) and KANK2 (kidney ankyrin repeat-containing protein) mutations: These are mutations associated with RHO GTPases, which are responsible for the regulation of actin remodeling and interfere with podocyte mobility [53]. This was extensively studied in rats. ARHGDIA and KANK2 mutation can cause a decrease in podocyte migration by interacting with Rho GTPase activity [20]. These patients can respond to eplerenone through modulation of Rac I-mineralocorticoid interactions [53].

Diagnostic criteria for steroid-resistant nephrotic syndrome

Like in any disease, it is of great importance the diagnostic criteria, in the case of SRNS, we first have to know what is the criteria of nephrotic syndrome diagnosis. Nephrotic syndrome diagnosis is based on one of various clinical factors: severe proteinuria (>1 g/m^2^/day), urinary protein creatinine ratio (UPCR) ≥ 200 mg/mmol (2 mg/mg) at first morning urine sample, or 3+ on urine dipstick plus either hypoalbuminemia (albumin <2.5 g/dL) or edema [18]. The majority of this type of illness in the pediatric population is considered as idiopathic nephrotic syndrome, the age of manifestation shows that approximately 6% manifest as congenital nephrotic syndrome, infantile nephrotic syndrome at ages three to 11 months is about 7%, from one to five year of age 51%, from six to 11 years 23%, and 13% at 12 years of age and older [54].

Knowing all this to diagnose a patient with SRNS, patients will always be treated first with oral prednisolone 2 mg/kg/day for four to six weeks followed by 1.5 mg/kg per dose on alternate days for another four to six weeks. During this time at Week 4 of treatment, it is advised to do a UPCR or three consecutive urine dipsticks, if results show a UPCR ≤20 mg/mmol or negative/trace dipstick, it is considered that the child has complete remission (SSNS). If none of this is achieved, this could be due to partial remission reason for which patients receive two additional weeks of treatment with or without three pulses of methylprednisolone (MPDN) and renin-angiotensin-aldosterone system inhibitors (RAASi). At Week 6 post-treatment if patients achieve remission, they are considered late responders (SSNS), but if no remission is seen during this time, patients are confirmed as SRNS [55].

In the two weeks of additional treatment for non-responders, genetic testing should be done to search for mutations in genes that encode podocyte proteins also a kidney biopsy to identify the underlying cause of the disease (most common histological diagnoses include focal segmental glomerulosclerosis, minimal change nephropathy, or mesangioproliferative GN) and study the degree of fibrosis in the glomeruli and renal interstitium. Biopsy should always collect a sample of >20 glomeruli, as FSGS lesions may be missed if the sample is not adequate [55-56].

It is also important to address secondary causes of NS in patients with SRNS such as metabolic or infectious causes. The main tests used for patients with SRNS are listed below (Table 1) [18,56].

**Table 1:** Clinical tests for children with steroid-resistant nephrotic syndrome Reprinted from "Management of steroid-resistant nephrotic syndrome in children and adolescents", by Tullus K, Webb H, Bagga A., 2018, Lancet Child Adolesc Health, 2(12):880-890

Main Tests	Additional Tests	Genetic Studies
Blood creatinine, electrolytes, protein, albumin, transaminases	Streptococcal antibodies, complement C3, C4 (if lupus nephritis is suspected)	Congenital (<3 months of age onset), infantile-onset nephrotic syndrome, family history of steroid resistance, suspected syndromic forms, resistance to calcineurin inhibitors, and before transplantation
Full blood count	Antinuclear antibody, anti-double-stranded DNA antibody (If lupus nephritis suspected)	
24-hour urine protein excretion, spot urine protein to creatinine ratio, or albumin to creatinine ratio	Hepatitis C and B serology, HIV antibody	
Varicella antibody status, measles immunoglobulin G (if no history of measles mumps and rubella vaccination)	Kidney biopsy: Light, immunofluorescence, and electron microscopy	

The importance of an adequate diagnosis for treatment is a key part of this disease, as studies have shown that there is a 50% risk of progression to end-stage kidney disease within the five years of diagnosis if partial or complete remission is not achieved. On the other hand, patients with FSGS who achieved complete remission had a five-year kidney survival rate of 90% [46-57].

Current treatment protocol for steroid-resistant nephrotic syndrome

After a correct diagnosis of SRNS, the next step is to use the most efficacious treatment, aiming to acquire complete remission of the disease if this is not possible then a partial remission is always acceptable [58-59]. An important part of the management of patients with SRNS is the first-line non-immunosuppressive treatment in children. This treatment consists of decreasing intraglomerular pressure, lowering proteinuria by 30%, and decreasing the progression of CKD [60]. In some cases, complete remission has been achieved with ACEi or ARBs plus PDN (prednisolone) therapy, and this therapy can be started after four weeks of initial PDN initiation [61]. The only disadvantage of this additional treatment is the increased risk of AKI and is even higher in patients with volume depletion or advanced CKD [62].

The first-line immunosuppressive treatments known for SRNS are Calcineurin Inhibitors with a vast amount of data known up to this moment for treatment with cyclosporine or tacrolimus, it's important to acknowledge that treatment with tacrolimus is preferred in some cases as it has shown lower relapsing risk, fewer side effects and even lower potential risk of nephrotoxicity compared to CSA treatment [6].

 Many studies have shown combining CNI (cyclosporine 4-6 mg/kg per day in two divided doses orally or tacrolimus 0.05-0.1 mg/kg in two divided doses orally) with steroids (oral PRED (40 mg/m^2^) given on alternate days until it was tapered and withdrawn after six months) have a great amount of success in remission and lowering the relapse rates in patients diagnosed with SRNS in the range of 30-80% [63-64]. This treatment has been compared with placebo, intravenous methylprednisolone, and mycophenolate mofetil with dexamethasone exhibiting superior results in the CNI with steroid groups [55]. In addition, studies have shown that long-term treatment with cyclosporine in children with SRNS reduces the progression to chronic kidney disease [65]. The Kidney Disease Improving Global Outcome guidelines recommend a continuation of CNI for a minimum of 12 months, supported by a tacrolimus study suggesting an 18-month treatment but despite these recommendations, an international agreement of the duration of treatment has not been established but most contemplate weaning CNI after two to three years [56,66].

The problem at the moment with this treatment is the nephrotoxicity caused by long-term use of CNI especially if eGFR decreases below 30 ml/min/1.73 m^2^. On biopsy of patients with steroid-dependent nephrotic syndrome, chronic lesions were reported in 35-75% caused by the use of CNI [66-68]. For this reason, treatment with CNI is not advised in patients with acute kidney injury, low eGFR, and/or uncontrolled hypertension; an exception is made in patients with chronic kidney disease if no other option is available for disease control, as CNIs may improve long-term kidney survival rate [55,69].

Treatment with mycophenolate mofetil is still controversial at the moment; a great reason for this is considered to be the lack of studies with statistical significance. At the moment, treatment with this drug is considered first in patients with CNI resistance to treatment, second patients who experience moderate or severe side effects with the use of CNIs, and third patients with eGFR below 30 ml/min/1.73m^2^ at a dose of 1200 mg/m^2^/d BID [25]. Also, the use of this medication is considered in patients with SRNS who have achieved full remission on CNI therapy for at least 12 months and discontinued CNIs or to maintain remission in pediatric patients with SRNS following CNI if they develop a steroid-sensitive relapse [55].

There is a debate on these treatment therapies in children with a confirmed mutation in podocyte genes, and many experts do not recommend this treatment, as the majority will not respond to it. An example is given in a study by Buscher et al. in which 91 children with SRNS were studied; 55% with non-genetic SRNS accomplished complete remission, 13% partial remission compared with 0% complete remission, and 29% partial remission in patients with genetic SRNS [14,70]. As for these patients, the only recommendation at the moment found is for supportive measures mostly because of a high chance of developing end-stage renal disease [18].

Role of biopsy and transplant in steroid-resistant nephrotic syndrome

The most common causes of renal failure in the pediatric population are hereditary diseases, nephrotic syndrome, and systemic diseases between the ages of five and 14 [71]. In nephrotic syndrome [18], a biopsy is done to determine histopathologic form causing nephrotic clinical features with or without renal failure and preventing remission by causing resistance to oral steroids after more than four weeks of treatment. Indications of biopsy are glomerular hematuria and proteinuria [72]. SRNS with histopathology of FSGS is at a higher risk of developing ESRD in patients.

The biopsy is an invasive procedure done via percutaneous insertion of the needle (either Tru-Cut needle or automatic spring-loaded biopsy gun) either blindly or via ultrasound guidance. The patient is kept in a prone position; the insertion is done at the lower lobe of the left side kidney in an attempt to avoid any vessel damage [72]. A biopsy is done to find the pathologic subtype behind the renal failure; SRNS is the most common indication for a biopsy (65.2%) [73], kidney biopsy is done when there is an unfavorable clinical course requiring diagnosis. The most common subtype is minimal change disease followed by focal segmental glomerulosclerosis [74]. A biopsy is diagnostic in 95.2% of children with renal failure of 4.8% [72].

A percutaneous biopsy is a low-risk procedure; most of the complications do not have any lifelong repercussions [75] and the most common complications are bleeding, hematoma formation, or infection [76]. After the procedure, the optimum observation period is 24 hours, anything less than eight hours would result in missing complications of the procedure [77]. Usual management of steroid-resistant nephrotic syndrome when end-stage renal disease (ESRD) has approached includes hemodialysis, peritoneal dialysis, and renal transplant. These are the attempts made to replace the role of the kidney in the body.

Transplantation is done in patients with rapidly progressing renal failure and ESRD. The FSGS subtype of SRNS is known to recur after kidney transplantation but that doesn’t mean that transplantation should be avoided in these patients because the recurrence rate is negligible. Transplant candidates are patients with SRNS; there is a chance of disease recurrence or renal allograft loss even after transplant. Disease recurrence occurs more in late steroid-resistant nephropathy (LSRN) than primary steroid-resistant nephropathy (PSRN). To avoid these post-transplant complications and organ failure, these patients are given targeted early intervention, i.e. aggressive plasmapheresis and immunosuppression post-transplant. The recurrence rate is also higher in patients with minimal change disease on histopathology compared to focal segmental glomerulosclerosis [78].

After a kidney transplant, the blood works show a sharp decline in creatinine and protein/creatinine ratio in an SRNS patient. The administration of an immunosuppressant (e.g. Rituximab) can help maintain the declined pattern of protein/creatinine, however, it has little or no effect on the creatinine [78]

Children with a history of recurrent kidney transplants are treated with prophylactic plasmapheresis; this regimen may have an added immunosuppressant if required. These patients are then considered for kidney transplants again. FSGS patients developing SRNS are more likely to have the above-explained clinical picture. These patients are monitored very closely immediately following the transplant. They follow a schedule of measuring proteinuria daily for a week; then, a weekly follow-up is done for the next four weeks, which then decreases to monthly follow-up, and then every three months, a follow-up is required before possible recurrence [79].

Post-transplant recurrence occurs mostly in the FSGS subtype SRNS. The patients who get a renal transplant done may need a second transplant, and around 55% of the patients with the second kidney transplant will have a relapse of SRNS [80]. In a patient with renal dysfunction, there is no benefit in keeping the patient on prolonged dialysis to attain more benefits from the transplant, so transplant should be done if needed by the patient once a human leukocyte antigen (HLA) matched donor's kidney is available [81].

Newer immunosuppressive agents for steroid-resistant nephrotic syndrome

Unfortunately, there is no "one size fits all" approach to treating nephrotic syndrome. Studies are being continuously done to find newer agents which have more targeted therapy and lesser side effects.

Rituximab is a chimeric anti-cluster of differentiation 20 (CD20) monoclonal antibody that has been used to treat several autoimmune diseases [82]. It helps by depleting B-cells by causing apoptosis. It may be helping by decreasing the interaction with regulatory T-cells, which help induce remission [83]. It has been shown efficacious in various studies to treat SSNS with varying results. However, there are limited data regarding its efficacy in SRNS. A case report on an 11-month boy showed complete remission when rituximab was used as the sole agent after failure with conventional treatment [84]. In another study, it was seen that rituximab helped in preventing recurrence post-transplantation. If therapy was started early, before the development of sclerosis, it was seen that rituximab could be considered a possible treatment [85].

Ofatumumab, another anti-CD20 antibody with a higher affinity to the CD20 molecule than rituximab, has also been studied for use in refractory NS. In a clinical trial, ofatumumab showed benefit to patients with rituximab-resistant SRNS [86]. While in another placebo-controlled clinical trial done on patients with multidrug-resistant NS, it was seen that all patients remained nephrotic after treatment with low-dose ofatumumab [87]. Larger RCTs are required to avoid publication bias and monitor the side effects related to B-cell depletion due to these drugs.

Abatacept is a synthetic analog of cytotoxic T lymphocyte antigen-4 (CTLA4), an antagonist of CD80 on T-cells. Podocyte injury is a mechanism for the development of nephrotic syndrome, and podocyte CD80 induction was found to produce podocyte foot effacement and proteinuria [88]. Varying case reports have been done on the response of abatacept in SRNS, but no common consensus is there regarding its efficacy [89-90].

## Conclusions

According to the International Study of Kidney Disease in Children (ISKDC), idiopathic nephrotic syndrome in children can be categorized depending on the response to steroids. Patients with SSNS are more likely to experience relapses and/or develop steroid dependence, both of which are difficult to treat. New research backs up the effectiveness of calcineurin inhibitors (CNIs) and mycophenolic acid in reducing SSNS relapses. Rituximab is also essential, but many issues about the initial dosage, course repeats, and long-term adverse effects remain unanswered. Chronic renal disease can result from SRNS, especially if it is resistant to therapy.

Medication with CNIs has improved the prognosis, and new evidence suggests that in many patients with complete remission, treatment can be stopped. Rituximab is less efficacious in CNI-unresponsive SRNS than in SSNS, and the function of other biologicals (such as ofatumumab, abatacept, and others) is unknown. Although some patients with partial or even complete responses to immunosuppression have been recorded, a large number of children with FSGS have hereditary origins, and the majority of patients do not react to immunosuppression. We recommend further studies and randomized control trials to evaluate treatments leading to long-term remission without maintenance immunosuppression in SSNS, in both genetic and immune-mediated SRNS.
